# Imaging of hip and thigh muscle injury: a pictorial review

**DOI:** 10.1186/s13244-019-0702-1

**Published:** 2019-02-15

**Authors:** Kolja M. Thierfelder, Judith S. Gerhardt, Ioan N. Gemescu, Susan Notohamiprodjo, Christoph Rehnitz, Marc-André Weber

**Affiliations:** 1 Institute of Diagnostic and Interventional Radiology, Pediatric Radiology and Neuroradiology, University Medical Center Rostock, Ernst-Heydemann-Str. 6, 18057 Rostock, Germany; 2 Department of Radiology and Medical Imaging, University Emergency Hospital Bucharest, Bucharest, Romania; 3 Department of Radiology, University Hospital, LMU Munich, Munich, Germany; 4 Diagnostic and Interventional Radiology, Heidelberg University Hospital, Heidelberg, Germany

**Keywords:** Muscle injury, Muscle tear, Magnetic resonance imaging, Soccer, Hip, Thigh

## Abstract

Muscle injuries of the hip and thigh are a highly relevant issue in competitive sports imaging. The gold standard in diagnostic imaging of muscle injuries is magnetic resonance imaging (MRI). Radiologists need to be familiar with typical MRI findings in order to accurately detect and classify muscle injuries. Proper interpretation of the findings is crucial, especially in elite athletes. In soccer players, muscle injuries of the hip and thigh are the most common reason for missing a game.

The present pictorial review deals with the diagnostic assessment, especially MRI, of muscle injuries of the hip and thigh. Typical MR findings in muscle injuries include edema, hematoma, and tendinous avulsion as well as partial or complete muscle tear. To estimate the time to return to play, a grading into three groups—muscle strain, partial tear, complete tear—has traditionally been used. Taking into account the most recent literature, there are other prognostic factors such as the longitudinal length of a tear, the tendon’s intramuscular component, or persisting edema.

## Introduction

Diagnosing and grading of muscle injuries are highly relevant issues in professional sports [1]. During training and matches, the musculoskeletal system of soccer players is exposed to complex biomechanical forces [2, 3]. As a result of muscle injuries, 37% of professional soccer players miss training or matches each season [4, 5]. In over 90% of cases, the muscles of the lower extremities are affected [5, 6]. Muscle injuries are not specific to certain sports, but there are some typical biomechanics and some injury patterns that occur more often in particular sporting activities (Table 1) [7, 8]. Among soccer players, about 35% of injuries are muscle injuries, which often occur during a match [5, 9]. In soccer, muscle injuries are the most frequent cause for missing a game [9, 10].

**Table 1:** Typical injuries for the most common sports [8]

Sport	Most common injuries
Soccer	Hamstring and quadriceps strain/cramps/contusions
Tennis	Rotator cuff tears, lateral epiconodylitis (“tennis elbow”)
Football	Lateral ankle sprain, ACL tear, ACC joint separation, hamstring strains
Basketball	Ankle sprains, ankle/foot fractures, knee joint injuries
Baseball	Rotator cuff tears, SLAP lesions, posteromedial elbow impingement
Volleyball	Acute ankle sprain, chronic/overuse knee injuries, shoulder overuse injuries
Rugby	Thoracic cage fractures, lower extremity muscle strains
Cycling	Acute trauma targeting any region (falling or traffic accident), anterior knee injury
Skiing	ACL/MCL sprains, femoroacetabular impingement, thumb ulnar collateral ligament sprain (skier’s thumb)

*ACL* anterior cruciate ligament, *ACC* acromioclavicular, *SLAP* superior labral tear from anterior to posterior, *MCL* medial collateral ligament

**Fig. 1:**
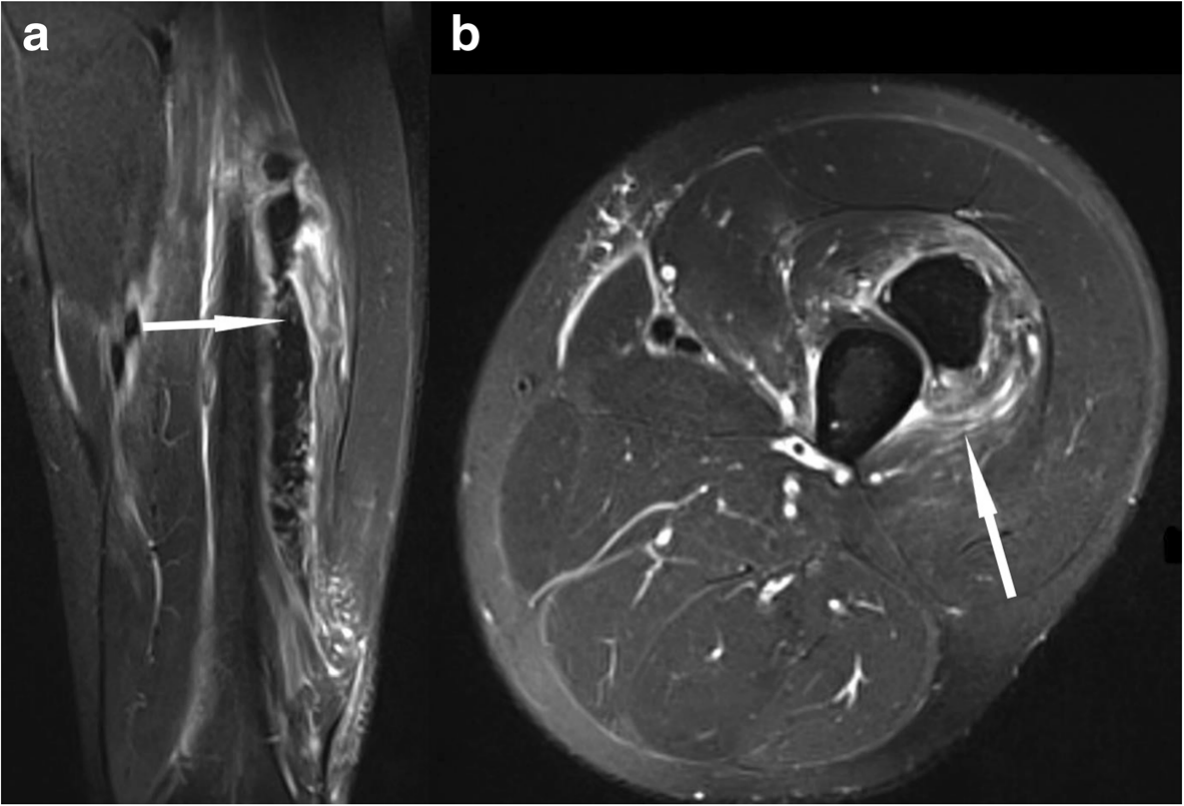
Coronal (**a**) and axial STIR (**b**). Feathery edema around hematoma within the M. vastus intermedius after blunt direct trauma

In the vast majority (around 96%) of muscle injuries, there is an indirect mechanism of accident, often with the result of a muscle tear [4]. In around 2%, a *direct* trauma is the reason for a muscle injury. In contrast to indirect injuries, there generally is a muscle contusion after direct trauma [11]. A blunt direct trauma that can induce a bleeding deep within the muscle, resulting in a hematoma, which is mostly clearly detectable by MRI (Fig. 1) [4]. Muscle contusions generally heal relatively fast, making return to play often possible within a week. In rare cases, direct muscle contusions can result in compartment syndrome [4, 9].

Unlike muscle contusions, muscle tears are generally caused not by a direct but an *indirect* injury. This indirect trauma is induced by eccentric contraction [12]. In MRI, typical features of indirect muscle injuries are hematoma, fiber disruption, and muscle edema. The muscles with a high risk for tears are types with a high proportion of type II fibers, muscles with multiple heads, and muscles extending over two joints like the M. biceps femoris, M. rectus femoris, and the adductor muscles [13]. There is also a higher risk for muscles that show high peak levels of stress in specific movements, e.g., the M. rectus femoris (during shooting) or the M. biceps femoris (during sprinting) [5]. In soccer players, the most commonly involved muscles are the hamstrings (37%), adductors (23%), and the quadriceps muscle (19%) [5].

The *localization* of a muscle tear depends on the age of the patient. In young athletes, the muscle typically tears at the non-fused apophysis. In elder people, the tear is most commonly located at the tendon, which is often already affected by degenerative changes. In adults of intermediate age, the location of the tear is often the myotendinous junction, which is the weakest link in the muscle-tendon-bone chain [7, 12].

From a radiological point of view, it is important to know the typical imaging findings and to be able to differentiate among the different kinds of muscle injuries. The prognosis varies widely among the different types, and there are many different therapy options.

### Imaging techniques

In muscle injuries of the hip and thigh, there is a large variety of imaging patterns, ranging from very subtle changes in muscle strains to complete tears with retraction of the muscle and large hematoma. Both ultrasound (US) and MRI are generally suitable to evaluate muscle injuries [14, 15]. US is often used as the first modality as it is widely available—even in proximity to the sports arena of several professional soccer clubs, fast, easy to use, and affordable. However, US is known to be highly user-dependent. In addition, the determination of the length of a muscle tear is difficult and small hematomas can be missed, especially within the first 24 h after injury. MRI is a more elaborate imaging technique that is considered the reference standard for muscle injuries. It allows for an excellent depiction of the most relevant findings in muscle injuries [6] and has a high sensitivity in identifying acute and chronic soft tissue alteration [16].

To assess muscle injuries on MRI, fluid-sensitive short-tau inversion recovery (STIR) or proton density fat-saturated (PDfs) sequences on the one hand and T1-weighted sequences on the other hand should be acquired as a minimum. Fluid-sensitive sequences allow to visualize edema, muscle tear, hematoma, and bone bruise. While PDf sequences provide more anatomical information, STIR is less sensitive to metal artifacts and patient movement. On the other hand, PD sequences have a higher signal to noise ratio (SNR) compared to STIR sequences. T1-weighted sequences allow for an assessment of (stress) fractures including bony avulsions and hematoma. Optionally, T2-weighted sequences can be used. Contrast-enhanced sequences are not necessary after acute trauma. Furthermore, it is a common practice to assess anatomical features on non-fat-suppressed images. Dixon sequences (chemical-shift imaging) can be useful as they provide separated water-only and fat-only images. This technique can provide a more uniform fat suppression [17, 18].

### Grading and prognosis

Muscle injuries in athletes are quite diverse and challenging to precisely define [4]. So far, a clear consensus on a *clinical grading system* does not exist. Every grading system has its strengths and weaknesses. The term muscle strain, though it is not consistently interpreted, is often used [4]. In order to determine the most suitable therapy and to estimate the time to return to play, a clinical examination is performed. It is generally based on a grading into three levels, considering bulge and loss of strength. One of the most commonly used clinical grading systems was introduced by O’Donoghue in 1962. Since then, several other muscle injury classifications were proposed, as presented in Table 2 [4].

**Table 2:** Clinical grading systems for muscle injuries

Name of classification	Year	Focus (if any)	Number of grades used
O’Donoghue	1962	–	3
Ryan	1969	Quadriceps (initially)	4
Takebayashi	1995	Ultrasound	3
Peetrons	2002	Ultrasound	3
Stoller	2007	MRI	3
Munich	2012	MRI	4 + contusions

**Fig. 2:**
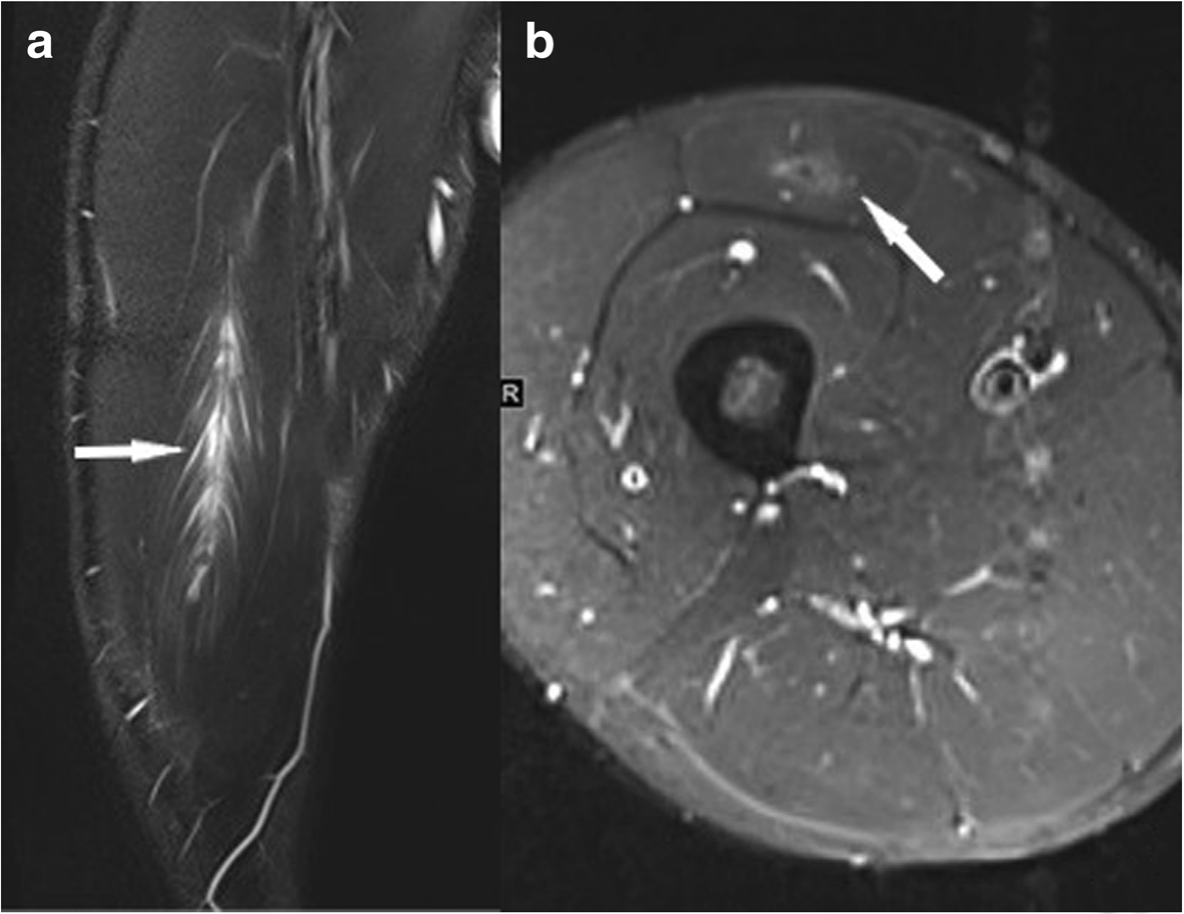
Coronal (**a**) and axial STIR (**b**). Grade 1 muscle injury of the M. rectus femoris with feathery intramuscular edema, but without muscle fiber discontinuity

**Fig. 3:**
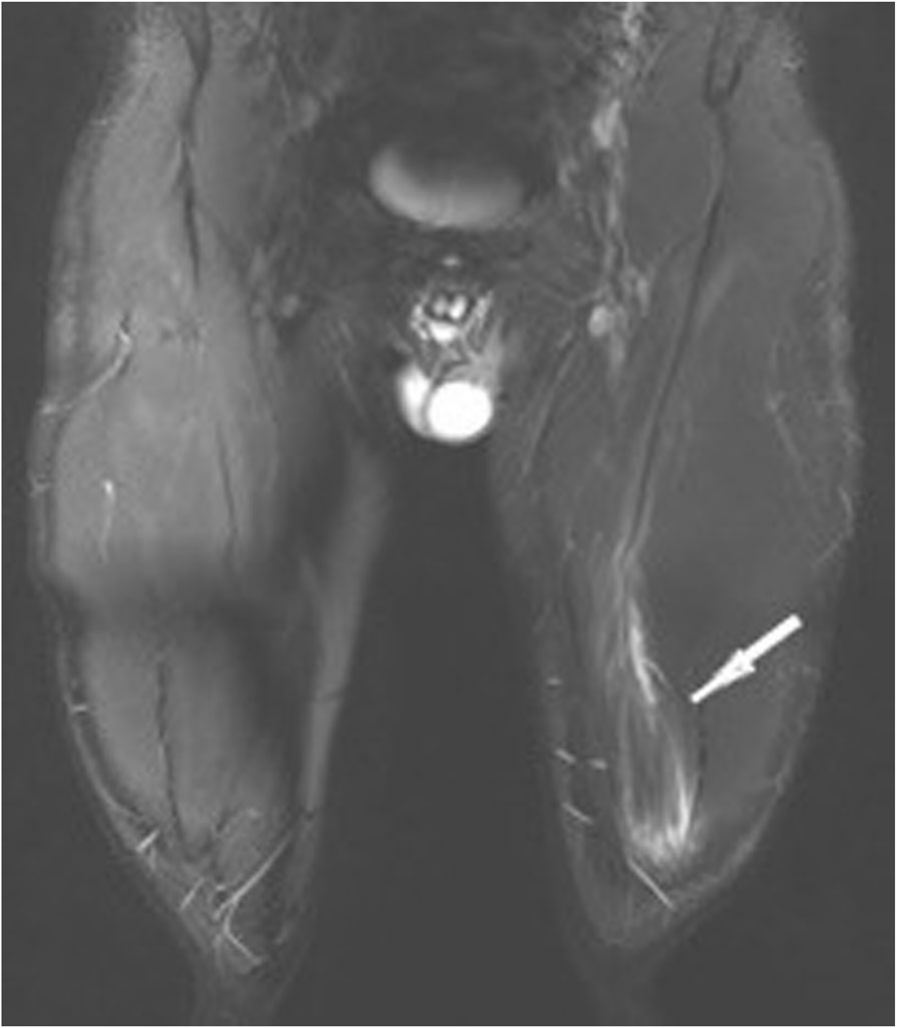
Coronal STIR sequence. Mild grade 2 injury with a partial tear of the M. rectus femoris with edema and hematoma

**Fig. 4:**
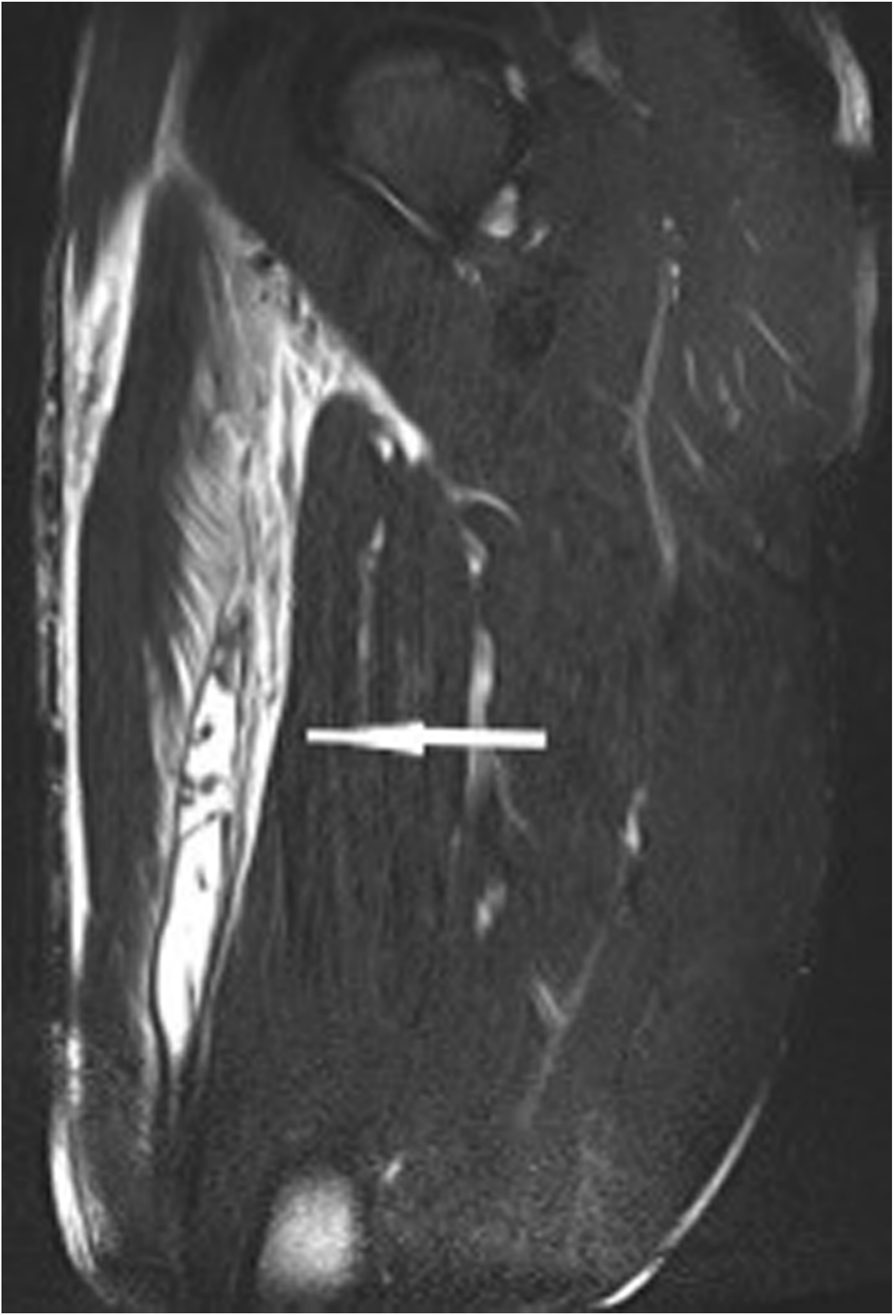
Sagittal T2w fat-saturated sequence. Severe grade 2 injury with a long distance subtotal tear of over 20 cm and hematoma of the M. rectus femoris

**Fig. 5:**
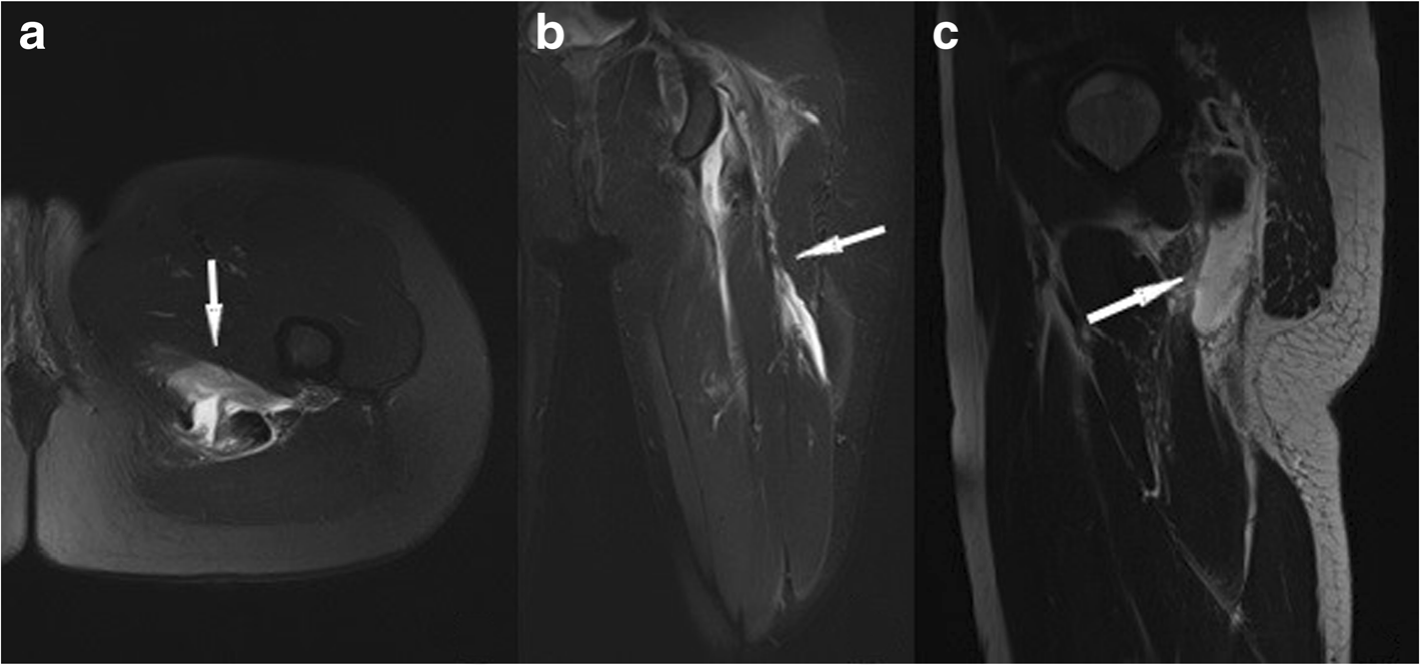
Axial PDfs (**a**), coronal PDfs (**b**), and sagittal T2w (**c**) images of a grade 3 injury of the hamstrings

The most common *MRI-based grading system* of muscle injuries, modified by the ultrasound-based Peetrons classification, distinguishes four grades [3] (Table 3). It provides only a rough estimate, and it suffers from the drawback of the wide range of grade 2 injuries and the non-existence of a rating for the tendon’s intramuscular component. Grade 0 is used in cases with a normal MRI. Grade 1 includes all cases with an MRI showing edema, but no structural damage or hemorrhage [4] (Fig. 2). Grade 2 is a partial and grade 3 a complete tear of the muscle (Fig. 3). The time to return to play is closely correlated to the grade—the higher the grade, the longer the time to return to play [3, 4]. The MRI correlate of a grade 1 muscle strain is an intramuscular hyperintensity on fluid-sensitive sequences without evidence of muscle fiber disruption (Fig. 2). The wide range of grade 2 injuries comprises tiny fiber discontinuities (Fig. 3) to large subtotal muscle ruptures (Fig. 4). A grade 3 injury represents a complete muscle tear (Fig. 5). In grade 3 injuries, the extent of muscle retraction needs to be determined and reported. Grade 3 injuries may require surgery.

**Table 3:** MRI-based classification system of muscle injuries and resulting lay-off time [3, 4]

Grade	MRI finding	Lay-off time (days)
0	Normal MRI	8 ± 3
1	Edema (Fig. 2)	17 ± 10
2	Partial tear (Figs. 3 and 4)	22 ± 11
3	Complete tear (Fig. 5)	73 ± 60

**Fig. 6:**
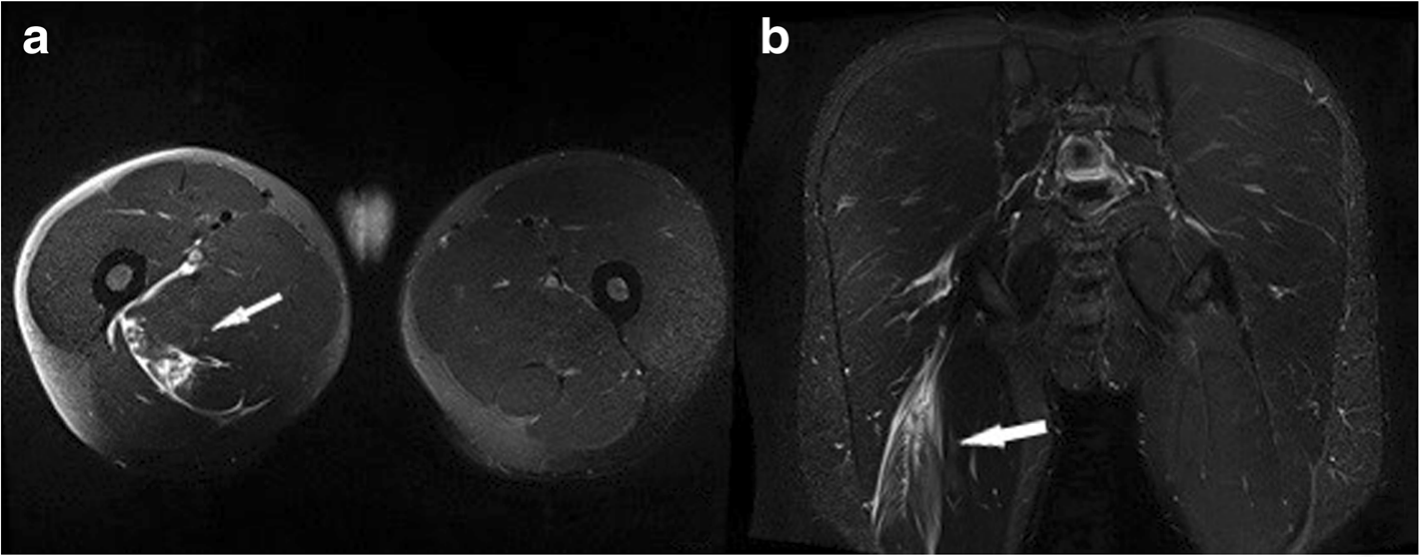
Axial T2 fs (**a**) and coronal STIR image (**b**) showing tears of the intramuscular component of the adductor and semitendinosus tendon

Further predictive factors for the lay-off time are the longitudinal length as well as proportion and volume of the muscle injury on MRI [6, 19]. The longitudinal length of the muscle injury, although it is not considered in the common grading system presented in Table 3, has the highest prognostic value. It reflects the number of muscle units that are separated from the aponeurosis [19]. The injury of the intramuscular component of the tendon (Fig. 6), which is not considered in the traditional grading system, is also associated with a delayed return to play [7, 20]. Moreover, a previous injury of a high-risk muscle increases the risk of another injury of the same muscle [21]. In elite soccer players, 16% of muscle injuries are re-injuries, which imply a 30% longer lay-off time [4]. An underestimated injury and the subsequent premature return to play is a common reason for recurrent muscle injuries. Also, an inadequate rehabilitation is a risk factor for muscle injuries in soccer players [9].

The common grading systems do not further divide the grades, so that injuries with a diverse etiology and prognosis are grouped together, especially in the heterogeneous group of partial tears. The Munich muscle injury classification is a comprehensive grading system for athletic muscle injuries with the aim of grading the injuries in a more comprehensive and comparable way by standardizing terms of muscle injuries. Indirect muscle injuries are classified into functional and structural disorders with sub-classification: functional disorders are defined as acute indirect muscle disorders *without* macroscopic evidence (in MRI or ultrasound) of muscular tear while structural disorders refer to any acute indirect muscle injury *with* macroscopic evidence (in MRI or ultrasound) of muscle tear [4]. Compared to functional muscle disorders, structural injuries are associated with longer time to return to play. This simple classification provides a good evaluation of injuries and helps to provide a proper diagnosis while reducing the chances of miscommunication and the rate of recurrence/complications. It has a strong correlation with the time to return to play [4].

### Differential diagnoses of muscle injuries of the hip and thigh

**Fig. 7:**
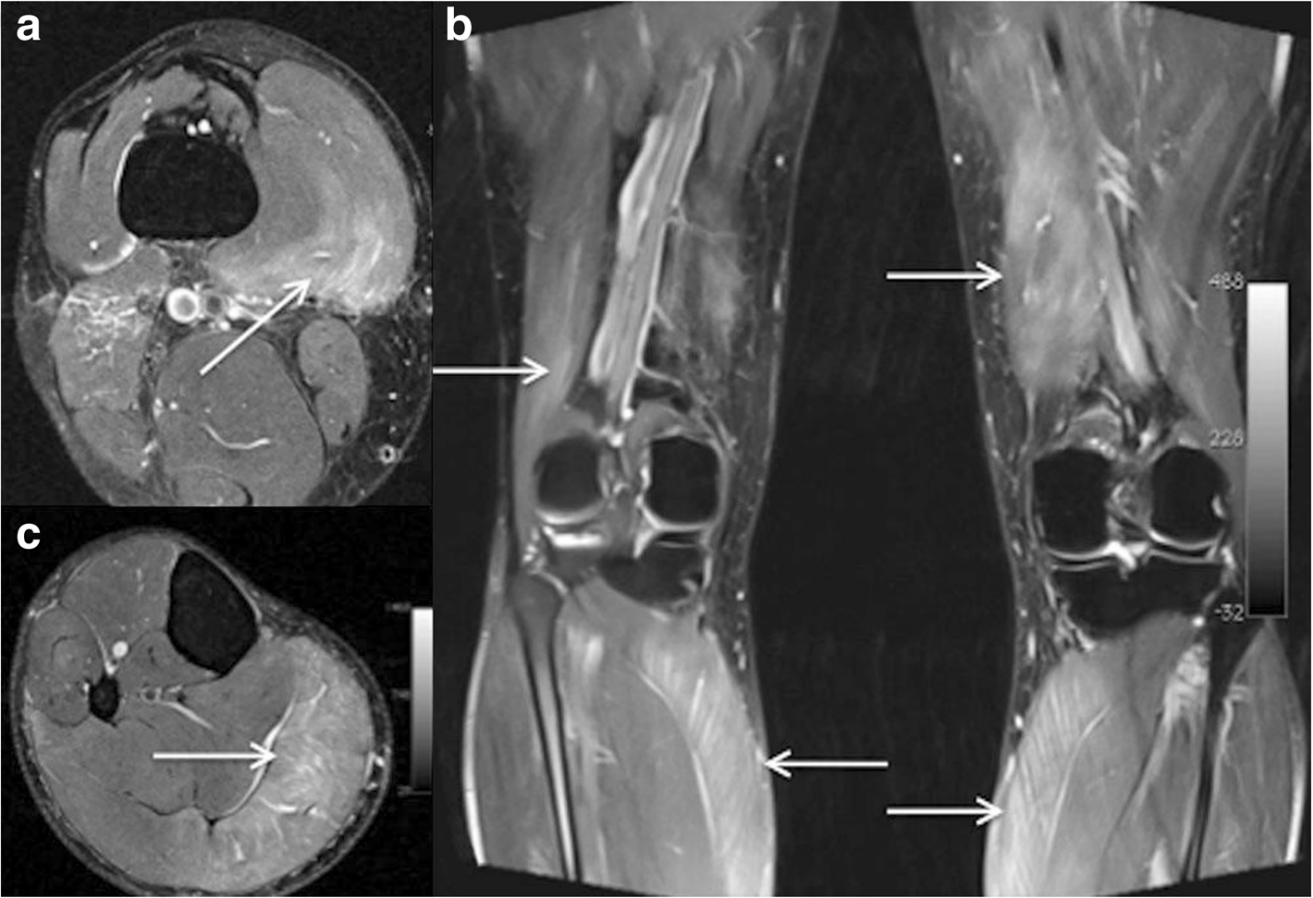
Axial (**a**, **c**) and coronal (**b**) PDf-weighted MR images in an athlete with delayed onset muscle soreness 4 days after completing a triathlon. There is an extensive lower limb edema bilaterally, predominately involving the vastus medialis and the medial head of gastrocnemius (arrows) (images with courtesy of Dr. Andrew Van Den Heever, Cape Town, South Africa). *Reprinted by permission from Springer: Springer Nature, Magnetic resonance imaging of the skeletal musculature, MRI of muscle injuries, Simon Dimmick, Christoph Rehnitz, Marc-André Weber, and James M. Linklater, 2014*

**Fig. 8:**
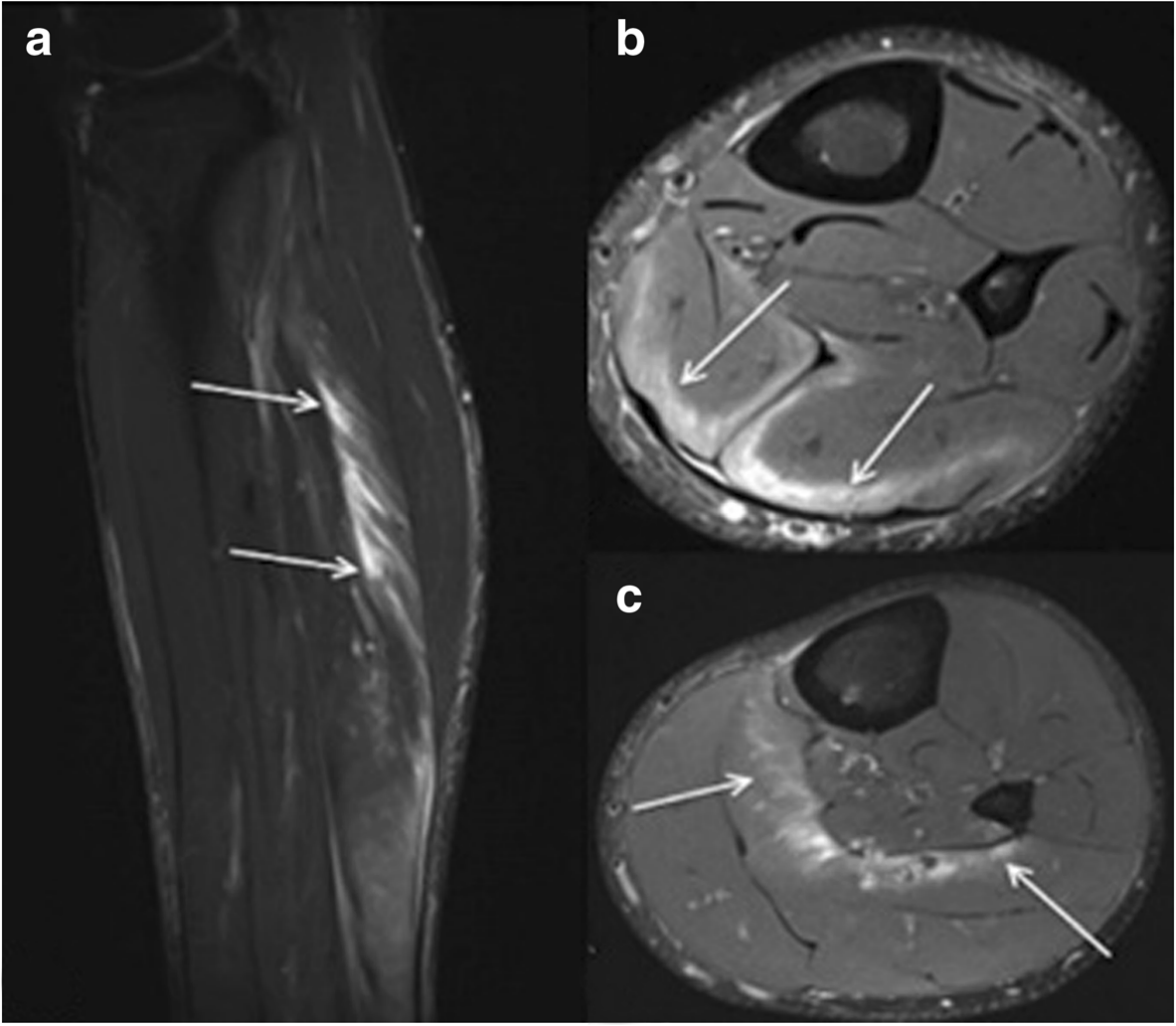
Sagittal STIR (**a**) and axial proton density-weighted MR images with fat saturation (**b**, **c**) showing extensive post-exercise edema within the soleus muscle in an Australian Rules football player. The edema is centered around the myotendinous junctions, demonstrating a geographically emarginated distribution (arrows). The patient played an entire game 2 days after this MRI. *Reprinted by permission from Springer: Springer Nature, Magnetic resonance imaging of the skeletal musculature, MRI of muscle injuries, Simon Dimmick, Christoph Rehnitz, Marc-André Weber, and James M. Linklater, 2014*

Differential diagnoses in MRI of muscle injuries are exercise-related delayed onset muscle soreness (DOMS) and post-exercise edema. *DOMS* occurs 1 or 2 days after exertion and manifests as local pain, soreness, and soft tissue swelling, often increasing over the course of 1 week (Fig. 7). The muscle appearance is similar to a grade 1 strain injury and shows a feathery-like pattern on fluid-sensitive sequences in relation to an inter-fascicular edema [22]. Unlike DOMS, the *post-exercise edema* usually resolves within few hours after exercise. As shown in Fig. 8, it can also appear similar to a grade 1 tear. Also, the IVIM sequence which will be mentioned below provides information on microvascular perfusion after muscle effort [23].

### Complications

**Fig. 9:**
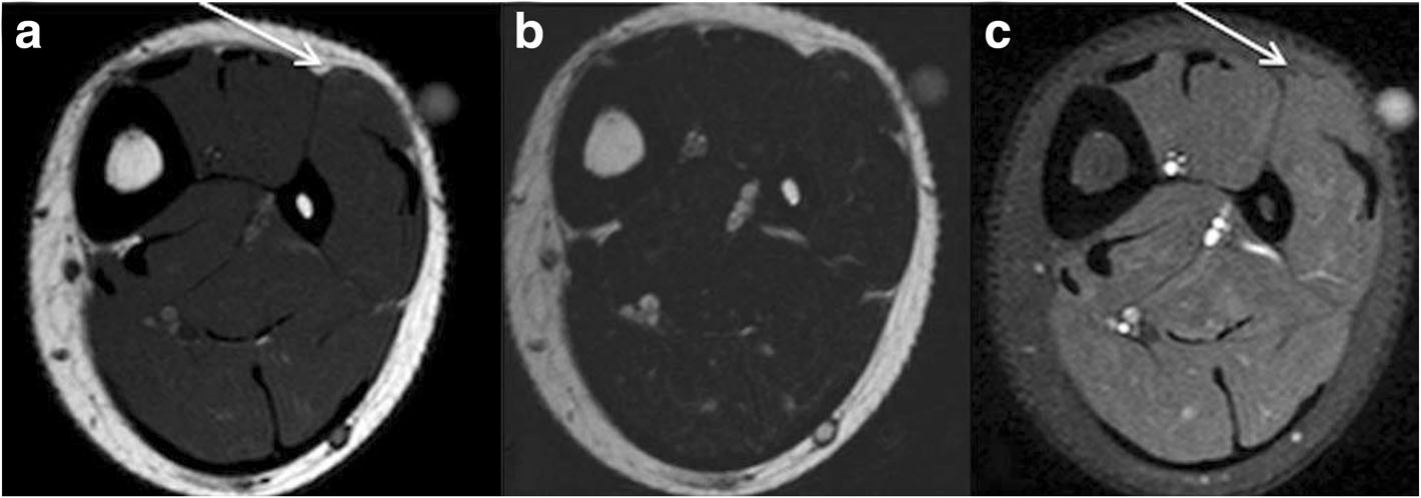
A 16-year-old kickboxer with a suspected “tumor” of the lateral leg, location marked with a capsule. Axial T1 (**a**), T2 (**b**), and post contrast T1f-weighted (**c**) MR images demonstrate a muscle hernia of the peroneus longus muscle with outpouching of the muscle through the antero-lateral investing fascia. The hernia follows the imaging characteristics of the muscle tissue in all sequences. There was a history of direct trauma while playing soccer 1 year ago, presumably resulting in a tear of the investing fascia. The patient noted an increase in size during training/muscle activity. *Reprinted by permission from Springer: Springer Nature, Magnetic resonance imaging of the skeletal musculature, MRI of muscle injuries, Simon Dimmick, Christoph Rehnitz, Marc-André Weber, and James M. Linklater, 2014*

Possible complications of muscle injuries are muscle hernia, acute or chronic exertional compartment syndrome, myositis ossificans, calcific myonecrosis, and Morel-Lavallée lesions [24]. *Muscle hernias* manifest in herniation of the muscle tissue through a small fascial defect in relation to a prior blunt or penetrating muscle trauma (Fig. 9). Muscle hernias increase in size during activity and can sometimes be indetectable during rest periods, thus making dynamic or standing ultrasound a requirement [25–28].

**Fig. 10:**
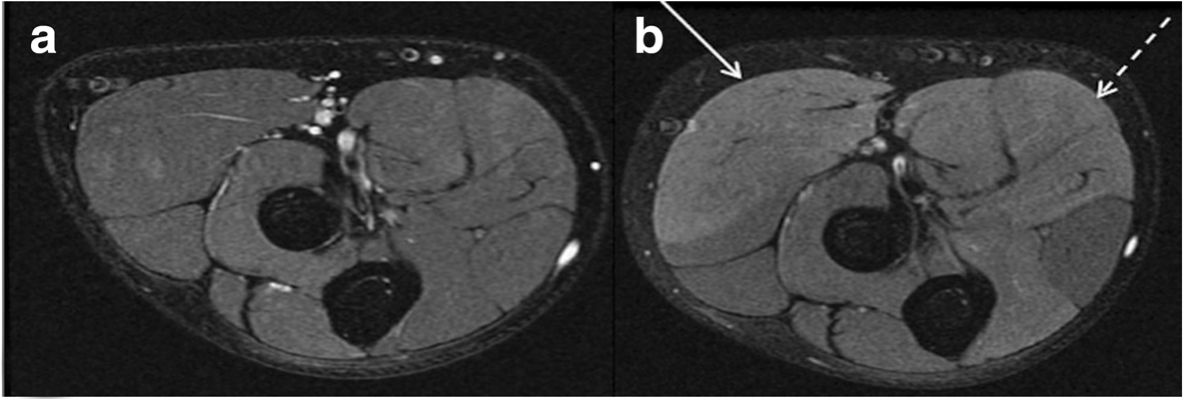
Axial proton density-weighted MR image with fat saturation. Exercise-induced compartment syndrome, (**a**) forearm prior to exercise, (**b**) post-exercise swelling and ground glass-like edema within the brachioradialis, and humeral head of pronator teres muscles (arrow), while the remainder of the flexor pronator muscle group appears normal, with the exception of flexor carpi ulnaris (dashed arrows). *Reprinted by permission from Springer: Springer Nature, Magnetic resonance imaging of the skeletal musculature, MRI of muscle injuries, Simon Dimmick, Christoph Rehnitz, Marc-André Weber, and James M. Linklater, 2014*

A *compartment syndrome* is caused by muscle anoxia by an increasing pressure within the compartment. The chronic exertional compartment syndrome results from an increased compartment pressure during exercise and resolves with rest. An acute compartment syndrome, on the other hand, is a surgical emergency presenting in with pain, which is disproportionate to the injury. On MRI, there are often bilateral signal hyperintensity peaks at exercise with delayed return to normal signal (Fig. 10).

**Fig. 11:**
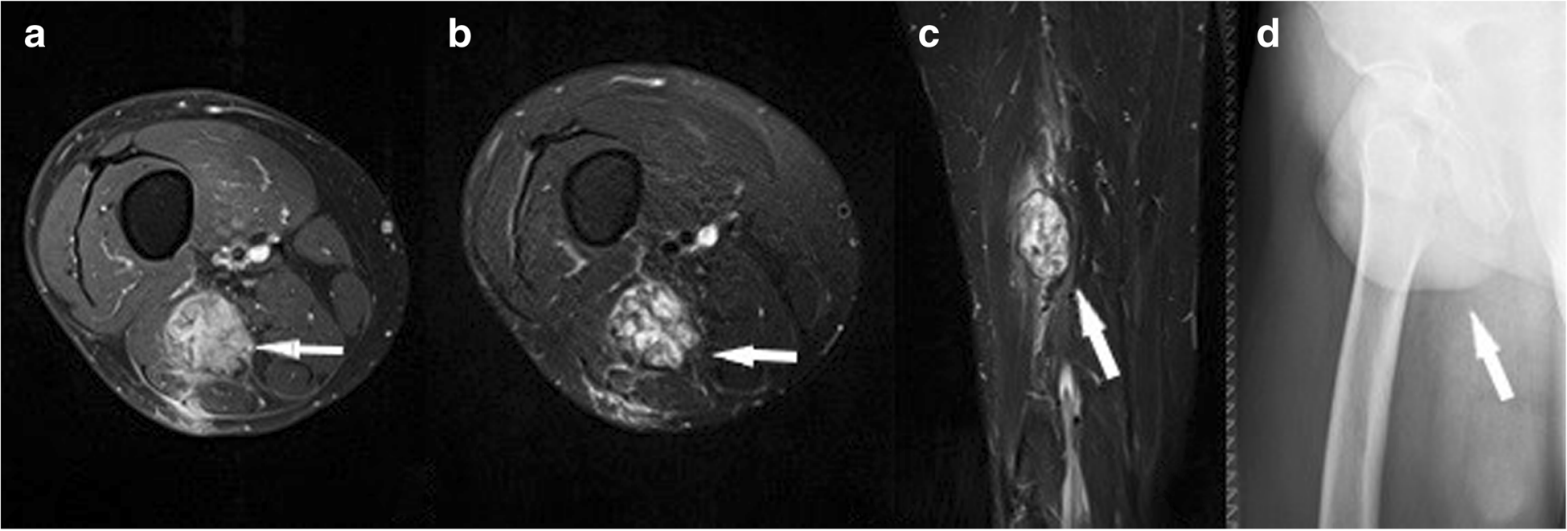
Axial T1fs with contrast medium (**a**), axial T2fs (**b**), coronal STIR (**c**), and standard radiograph (**d**), depicting myositis ossificans in the hamstrings

*Myositis ossificans* (better: heterotopic ossification, as the condition is not an inflammatory process) is a common sequelae of muscle injuries. Initially, hematoma and edema are present. On MRI, there is a high signal intensity on fluid-sensitive images and an increased enhancement after contrast administration. After about 6 weeks, typical imaging features with a peripheral rim of ossification that progresses towards the center can be found [25] (Fig. 11).

**Fig. 12:**
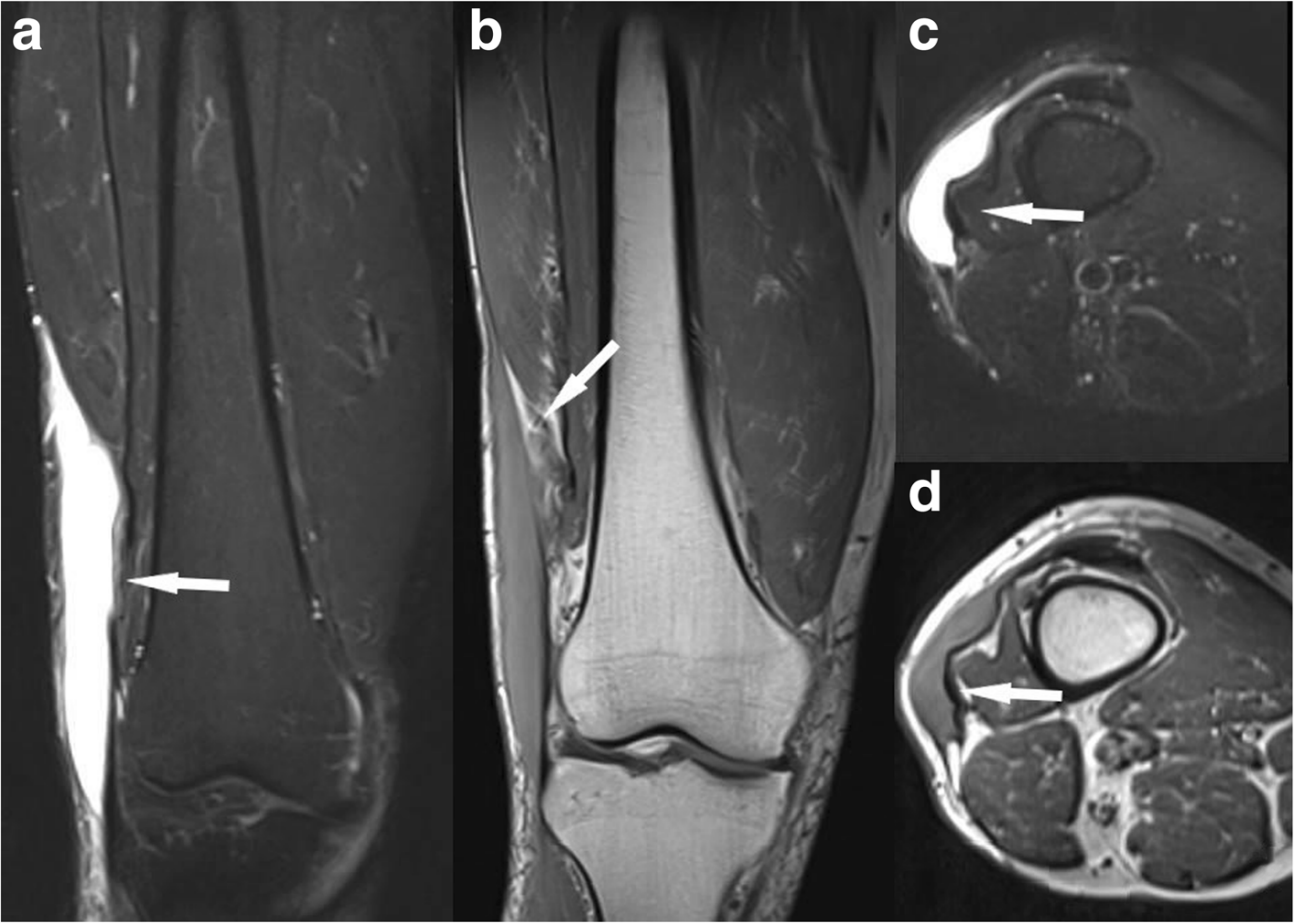
Coronal PDfs (**a**) and T1 (**b**), axial PDfs (**c**), and T1 (**d**). Morel-Lavallée lesion of the distal thigh

*Morel-Lavallée lesions* result from a closed degloving injury as a result of shear forces associated with a serve trauma and resulting separation of the subcutaneous tissue from fascia [29]. On MRI, it is often accompanied with recurrent fluid accumulation, while the fluid is of variable signal intensity (Fig. 12).

### Research on muscle injuries

MR techniques that are used as research tools in the assessment of the skeletal muscles are blood oxygenation level-dependent (BOLD), diffusion tensor imaging (DTI), T2 mapping, and intravoxel incoherent motion (IVIM) [14, 23, 30, 31]. These techniques allow for non-invasive functional assessment of peripheral microvasculature in the skeletal muscles. While these techniques have currently no role in clinical routine, they improve the understanding of muscular and vascular physiology and alterations of microcirculation.

At *BOLD MRI*, which is founded on the different magnetic properties of oxy- and deoxyhemoglobin, blood is used as an endogenous contrast agent. BOLD allows to depict micro-vascularization, metabolism, and vascular insufficiency as in compartment syndrome [7, 14, 32]. In skeletal muscles, the BOLD contrast is generated by the microcirculation yielding that is very sensitive for alterations of the physiological oxygen supply and demand [7].

*DTI* of the muscle tissue allows examining the microarchitecture, the extraction of diffusion indices, and fiber tracking. DTI assesses integrity and orientation of muscle fibers. Changes at microscopic level like *z*-band disruptions can be detected. To monitor the conditions of the skeletal muscle, DTI is evolving to a clinical feasible tool and it may help to understand the pathophysiology in chronic exertional compartment syndrome [30].

*T2 mapping* detects muscle activation in specific groups and early fatty atrophy. During and after activity, T2 increases and the recruitment and capacity can be determined. T2 mapping may detect early microscopic fatty atrophy, not detected with morphologic MRI, represented as increased T2 values [14, 32]. Other advanced MR imaging techniques for muscle injuries are MR spectroscopy measuring the muscle energy and lipid metabolism and ASL detecting the blood flow within the muscle tissue.

IVIM extracts information regarding microvascular blood flow out of multiple *b* value diffusion acquisitions. It can determine an increase in microvascular perfusion in a specific segment after a particular task and can correlate this perfusion with the duration of the effort. Also, this method has shown to be able to quantify microvascularity and microstructure, evaluating the depletion of the capillaries and the degradation of myofibers, thus useful in the evaluation of dermatomyositis [23, 31].

## Conclusion

The reference standard of imaging muscle injuries of the hip and thigh is MRI using fluid-sensitive and T1-weighted sequences. Typical findings are edema, hematoma, and partial or complete muscles tears. Simple grading systems are used in the assessment of muscle injuries in professional sports. However, specific imaging features such longitudinal length and volume of the muscle tear and involvement of the intramuscular component of the tendon have recently shown a significant impact on prognosis. These features should be precisely described, and a close collaboration with the team doctor is very helpful.

In order to optimally support athletes and team doctors, radiologists should be aware of the most common differential diagnoses delayed onset muscle soreness and post-exercise edema and complications like heterotopic ossification, compartment syndrome, or muscle herniation.
